# Clinical and serological evaluation of capybaras (*Hydrochoerus hydrochaeris*) successively exposed to an *Amblyomma sculptum*-derived strain of *Rickettsia rickettsii*

**DOI:** 10.1038/s41598-020-57607-5

**Published:** 2020-01-22

**Authors:** Alejandro Ramírez-Hernández, Francisco Uchoa, Maria Carolina de Azevedo Serpa, Lina C. Binder, Alessandra Castro Rodrigues, Matias P. J. Szabó, Andrea Fogaça, Celso Eduardo Souza, Marcelo B. Labruna

**Affiliations:** 1 Department of Preventive Veterinary Medicine and Animal Health, Faculty of Veterinary Medicine, University of São Paulo, Av. Prof. Orlando Marques de Paiva 87, São Paulo, SP 05508-270 Brazil; 2 Reference Rickettsial Diseases Laboratory, Superintendence for Control of Endemic Diseases, Mogi Guaçu, SP Brazil; 3 Ixodology Laboratory, Faculty of Veterinary Medicine, Federal University of Uberlândia, Uberlândia, MG Brazil; 4 Department of Parasitology, Institute of Biomedical Sciences, University of São Paulo, São Paulo, SP Brazil

**Keywords:** Infectious diseases, Biological techniques

## Abstract

Brazilian spotted fever (BSF), caused by *Rickettsia rickettsii*, is the most lethal tick-borne disease in the western hemisphere. In Brazil, *Amblyomma sculptum* ticks are the main vector. Capybaras (*Hydrochoerus hydrochaeris*), the largest living rodents of the world (adults weighing up to 100 Kg), have been recognized as amplifying hosts of *R. rickettsii* for *A. sculptum* in BSF-endemic areas; i.e., once primarily infected, capybaras develop bacteremia for a few days, when feeding ticks acquire rickettsial infection. We conducted experimental infections of five capybaras with an *A. sculptum*-derived strain of *R. rickettsii* and performed clinical and bacteremia evaluation during primary and subsequent infections. Bacteremia was detected in all capybaras during primary infection, but not in subsequent infections. All animals seroconverted to *R. rickettsii* (titres range: 64–32,768), and remained seropositive throughout the study. Primary infection resulted in clinical spotted fever illness in four capybaras, of which two had a fatal outcome. Subsequent infections in seropositive capybaras resulted in no clinical signs. Capybaras developed a sustained immune response that prevented a second bacteremia. This condition may imply a high reproduction rate of capybaras in BSF-endemic areas, in order to continuously generate capybaras susceptible to bacteremia during primary infection.

## Introduction

The bacterium *Rickettsia rickettsii* is the etiological agent of Rocky Mountain spotted fever, also known in Brazil as Brazilian spotted fever (BSF), a disease that has been registered in different American countries including Canada, United States, Mexico, Costa Rica, Panama, Colombia and Argentina^1,2^. This bacterium is transmitted by different tick species throughout Americas [i.e., *Dermacentor variabilis*, *Dermacentor andersoni*, *Rhipicephalus sanguineus* sensu lato (s.l.), *Amblyomma cajennense* species complex, and *Amblyomma aureolatum*]^2^. In Brazil, in the southeastern region, *Amblyomma sculptum* (a member of *A. cajennense* species complex) is the main incriminated vector, for which capybaras (*Hydrochoerus hydrochaeris*) and horses act as primary hosts for all parasitic stages^3,4^.

BSF is the most lethal tick-borne disease in Brazil with increasing numbers of cases and deaths. Between 2007 and 2015, 17,117 suspected cases of spotted fever (including other spotted fever group rickettsioses) were reported and 1,245 were confirmed as SFG rickettsioses in 12 Brazilian states from all regions^1^. Moreover, case-fatality rates have attained values of 30% or higher, which could be associated with low index of suspicion and misdiagnosis by health-care professionals and exposure to particular eco-epidemiological risk factors^1^.

Ticks function as the main reservoirs of *R*. *rickettsii* in nature, nonetheless, due to bacterial pathogenic effects on ticks^5,6^ and some degree of tick refractoriness to bacterial infection^7,8^, low natural infectious rates (<1%) are commonly found in endemic areas. Therefore, amplifying hosts (vertebrate animals developing bacteremia for some days) are essential in maintaining the pathogen by its transmission to non-infected ticks and creating new cohorts of infected ticks within the population^3,9^. In Brazil, opossums (*Didelphis aurita*)^10^ and capybaras^11,12^ were shown to be competent amplifying hosts of *R*. *rickettsii* for *A*. *sculptum* ticks, in contrast to horses^13^. However, in the two previous studies performed with capybaras^11,12^, authors used *R. rickettsii* strains that were not of capybara or *A. sculptum* origin, which could have resulted in less realistic results. In addition, it was never tested if a capybara could develop bacteremia after a primary infection, a condition of great importance for the epidemiology of BSF.

In the present study, we performed multiple exposures of capybaras with a *R. rickettsii* strain derived from a capybara-associated tick vector (*A. sculptum*). We aimed to evaluate the clinical and serological profile of these capybaras during primary and subsequent infections via tick parasitism, and the occurrence of bacteremia during all infection challenges.

## Materials and Methods

### Animals

Five capybaras (no. 1 to no. 5) were obtained from two different BSF-nonendemic areas in São Paulo state. Three females (no. 1, 2, 3) from São Paulo city [University of São Paulo (USP) campus], 2 weeks old and from the same litter; and, one male and one female (no. 4 and 5) from Pirassununga city (USP campus), 4 weeks old from two different groups. Animals were transported to the Animal Research Facility of Superintendência de Controle de Endemias (SUCEN) in Mogi Guaçu, São Paulo state. They were kept in individual boxes (3 m × 3 m) and fed daily with fresh forage, commercial guinea pig pellets and water *ad libitum*. Occasionally, they received sugar cane and fresh corn as positive reinforcements during management. For environmental enrichment, capybaras had permanent access to a swimming pool, with daily cleaning and water replacement. All animals had been naturally exposed to *Amblyomma dubitatum* ticks before being transported to the Animal Research Facility.

Laboratory guinea pigs (Hartley strain) and white New Zealand rabbits were purchased from a commercial breeder and housed in standardized laboratory cages. These animals were fed with a commercial guinea pig and rabbit pellet diet, had water *ad libitum* and were kept in facilities with temperature, photoperiod (12/12 h) and ventilation control. All guinea pigs and rabbits were tick-naïve and bred under laboratory standard sanitary conditions.

All animal procedures were authorized by the Ethics Committee on Animal Use of the Faculty of Veterinary Medicine of the University of São Paulo (CEUA project No. 4115110215), and procedures involving capybaras were authorized by the Brazilian biodiversity agency SISBIO (“Sistema de Autorização e Informação em Biodiversidade”-ICMBio) (License No. 43259-3). All methods were performed in accordance with the guidelines and regulations of the Brazilian National Council of Animal Experimentation (CONCEA).

### Capybara infection

**Figure 1:**
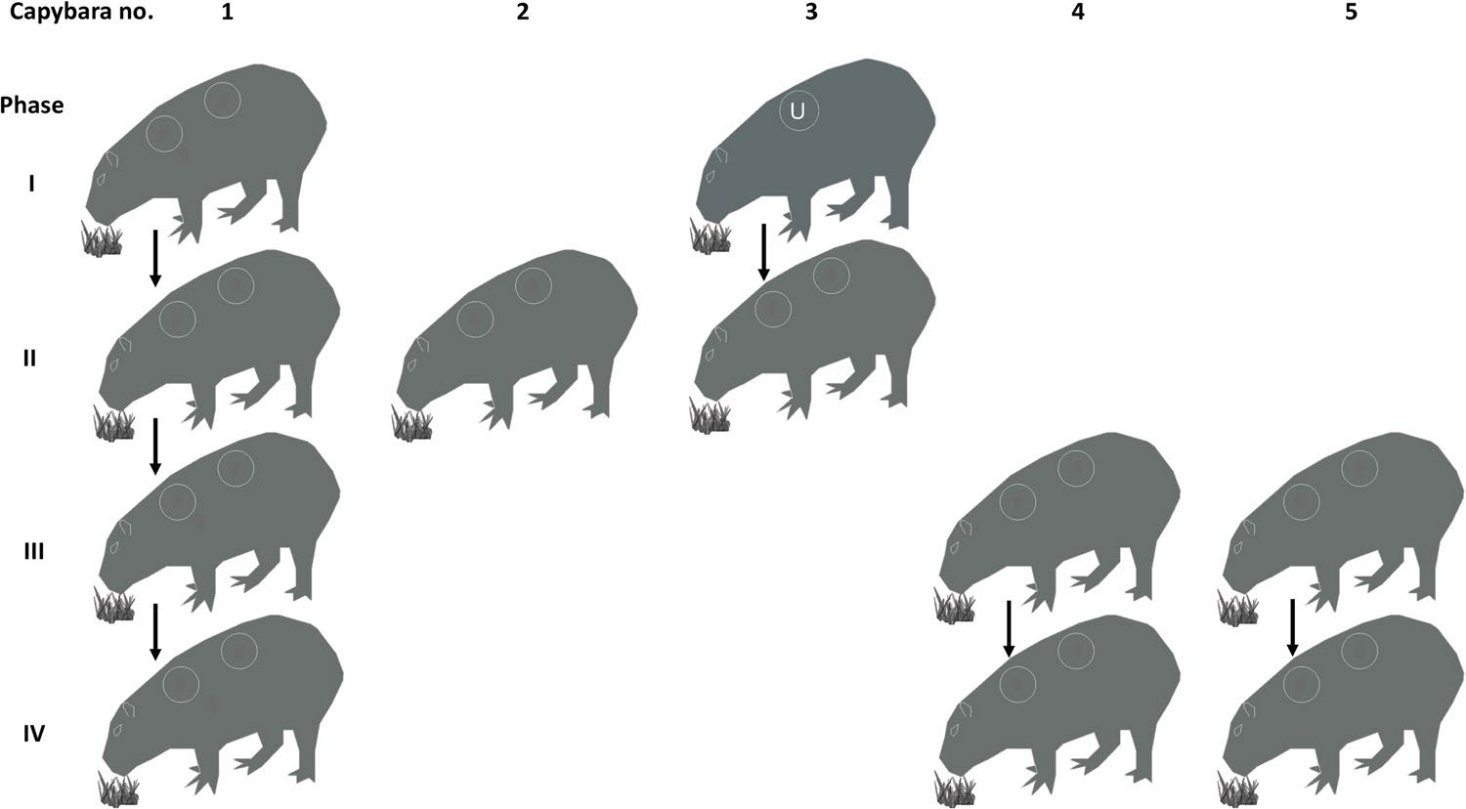
Scheme of the experimental infections conducted on capybaras no. 1 to 5 during the study. Phase I, primary infection of capybara no. 1, and retaining capybara no. 3 as noninfected control; phase II, second infection of capybara no. 1 and primary infection of capybaras no. 2 and 3; phase III, third infection of capybara no. 1 and primary infection of capybaras no. 4 and 5; phase IV, fourth infection of capybara no. 1 and second infection of capybaras no. 4 and 5.

The study was divided into four phases (I to IV) as follows: phase I, primary infection of capybara no. 1, and retaining capybara no. 3 as noninfected control; phase II, second infection of capybara no. 1 and primary infection of capybaras no. 2 and 3; phase III, third infection of capybara no. 1 and primary infection of capybaras no. 4 and 5; phase IV, fourth infection of capybara no. 1 and second infection of capybaras no. 4 and 5 (Fig. 1). At the moment of primary infection, capybara no. 1 was three months old (weight: 9 Kg), capybaras no. 2 and 3 were seven months old (weight: ≈14 Kg), and capybaras no. 4 and 5 were five months old (weight: ≈13 Kg).

Before primary infection, all capybaras were clinically healthy and the indirect immunofluorescence assay (IFA) with paired serum samples taken at a 14-day interval indicated that they were serologically non-reactive (serum dilution 1:64) to crude antigens of *R*. *rickettsii*, *Rickettsia parkeri*, *Rickettsia rhipicephali*, *Rickettsia bellii*, *Rickettsia typhi* and *Rickettsia felis*, as previously described^12,14^.

For primary and subsequent infections of capybaras, two separated cotton sleeves (10-cm diameter feeding chambers) were glued on the capybara shaved dorsum, as previously described^12,15^. These chambers were labelled as chamber A (cranial position on the capybara dorsum) and chamber B (caudal position on the capybara dorsum).The minimum distance between the two chambers was 5 cm. In all cases, chamber A received 20 males and 20 females (except for the primary infection of capybara 1, which consisted of 10 males and 10 females) of *A*. *sculptum* from a *R. rickettsii-*infected colony. These adult ticks derived from the fifth generation (F_5_) of a tick colony that was established in the laboratory from a population adapted to capybaras, collected in Itu municipality (São Paulo) in 2012^16^. These adult ticks were previously exposed to rickettsial infection as larvae and nymphs through feeding on guinea pigs in bacteremia that were intraperitoneally inoculated with *R*. *rickettsii* strain Itu, as described^8^. This rickettsial strain was isolated from *A*. *sculptum* ticks collected in the same area of origin of the progenitor ticks that generated our tick colony^16^. The infection rates were ≈30% for the tick batch used in phase I and 50–60% in the batch used during phases II and III (capybaras 2–5). This rate was not calculated in phase IV due to a limited number of available ticks in the colony. Chamber B received uninfected ticks and the results from this chamber will be presented in a subsequent manuscript.

Day 0 (zero) of infection was considered as the day of infestation with infected ticks for the first time (primary infection). Capybara no. 1 were infested again with infected ticks at 120, 248, and 475 days post primary infection (DPI), giving a total of four infection challenges. Capybaras nos. 2 and 3 were each exposed once to infected ticks, whereas capybaras no. 4 and 5 had an additional exposure to infected ticks at 227 DPI (total: two infection challenges). In each infestation with infected ticks, animals were monitored daily during 30 continuous days, for fever (rectal temperature) and other clinical signs. Also, blood samples were collected, within the 30-day following period, from the femoral vein, for the following procedures: (i) with anticoagulant (EDTA) for guinea pig inoculation (every 2 days), DNA extraction (every 2 days) and hematology (every 4 days); and (ii) without anticoagulant to obtain sera for IFA test (every 6 days). In addition, a skin biopsy from the abdominal region was performed with a 5 mm-punch, every 2 days (within the 30-day following period). These procedures were repeated during all study phases. In the control animal (capybara no. 3 in phase I), only blood was collected for hematology analyses. Prior to each procedure (sample collections and tick feeding chamber preparation) animals were slightly sedated with a mixture of xylazine (0.1 mg/kg) and ketamine (1 mg/kg) by the intramuscular route (IM).

In each infection phase (I to IV) a tick-naïve white New Zealand rabbit was used as control of infected ticks. At the same day of capybara infestation with infected ticks, these rabbits received the same number and batch of infected adult ticks. Formerly, a cotton sleeve (8-cm diameter feeding chamber) was glued on the shaved back of the rabbits as described earlier^17^. Clinical signs and rectal temperature were registered daily, between 0 and 21 days post infestation, and in case of death, necropsy and organ samples (spleen) were collected and frozen. Also, serum samples were collected in a 21-day interval for IFA (described below).

### Guinea pig inoculation

Anticoagulated blood from capybaras (1.0 ml) was intraperitoneally inoculated into two guinea pigs, for each blood sampling, in order to identify a probable bacteremia in the capybara, as previously described^12^. For this purpose, guinea pigs were previously anesthetized with a mixture of xylazine (10 mg/kg) and ketamine (100 mg/kg) (IM)^18^ and a blood sample was collected by intracardiac puncture for IFA analyses (day 0). The clinical signs and rectal temperature of all animals were monitored daily during 21 days. A second blood sample was collected at day 21, as described above, and thereafter guinea pigs were euthanized with sodium pentobarbital (100 mg/kg) by intracardiac injection. A guinea pig was considered febrile if rectal temperature was ≥40 °C for at least two consecutive days. Dead individuals were submitted to necropsy and samples of spleen were frozen at −20 °C for further DNA extraction and real-time PCR for *Rickettsia*.

### Hematology tests

Whole blood samples from capybaras (0.5 ml) were used for estimation of packed cell volume (PCV), red blood cells (RBC) and white blood cells (WBC) total count. PCV was estimated by the microhematocrit technique and red and white cells were counted in blood diluted with Gowers and Türk solutions, respectively, using an improved Neubauer chamber, following the procedures described by Madella *et al*.^19^.

### Serology: Indirect immunofluorescence assay (IFA)

Sera obtained from capybaras, guinea pigs and rabbits were tested by IFA using *R*. *rickettsii* (strain Taiaçu) crude antigen and fluorescein isothiocyanate-labelled sheep anti-capybara IgG (CCZ, São Paulo, SP, Brazil), goat anti-rabbit IgG (Sigma, St. Louis, MO, USA) and rabbit anti-guinea pig IgG (Sigma), respectively, as previously described^7,8,14^. Samples were initially tested in a 1:64 dilution (with PBS) as cut-off and those reactive, further diluted in twofold increments to the endpoint titre, as reported earlier^20^. In each slide, previously known reactive and non-reactive serum, for each animal species (capybara, rabbits or guinea pig) were included as positive and negative controls, respectively.

### Real-time PCR analyses of blood and tissue samples

DNA from frozen (−20 °C) blood (capybaras), skin (capybaras) and spleen (guinea pigs and rabbits) samples was extracted using the DNeasy Blood and Tissue kit (Qiagen Inc., Valencia, CA, USA), following the manufacturer’s protocols. Final products were stored at −20 °C for further amplification by polymerase chain reaction (PCR). Extracted DNA samples were used as template to amplify a 147-bp fragment of the citrate synthase gene (*gltA*) of *Rickettsia* spp. by a TaqMan real-time quantitative PCR using the primers CS-5 (forward^21^) and CS-6 (reverse^22^) and an internal fluorogenic probe (6-FAM d, BHQ- 1) (Integrated DNA Technologies, San Diego, CA), in accordance with reagents and cycling conditions previously reported^22^. The sensitivity of the technique was determined to be 1 DNA copy of *R. rickettsii*^22^. For each reaction, a positive (DNA of *Rickettsia vini* cultivated in Vero cells) and a negative control (molecular-grade water) were included.

The capybara blood and skin PCR-positive samples were submitted to a second qPCR analysis using the methodology described for the first qPCR. The absolute number of rickettsiae per mL of blood or per mg of tissue was determined using a standard curve established with the cycle of quantification (Cq) values of reactions using a dilution series of 10^2^ to 10^7^ copies of a plasmid (pGEM-T Easy, Promega, Madison, USA) containing the 147 pb fragment of *gltA*. All samples were analyzed in three technical replicates and respective means were calculated.

### Histopathology and immunohistochemistry

Standard histopathology sections from organs of capybaras that died from *R. rickettsii* infection were cut from formalin-fixed tissues (immersed in 10% neutral buffered formalin for 24 hours and in 70% ethanol afterwards). Then they were embedded in paraffin and stained with hematoxylin-eosin. Immunoalkaline phosphatase staining using naphthol fast red substrate, hematoxylin counterstain and a polyclonal rabbit anti-*R. rickettsii* antiserum (Adolfo Lutz Institute, São Paulo, SP, Brazil) was used for immunohistochemical testing for spotted fever group rickettsiae.

## Results

### Capybara infection

Five capybaras were infected during the four study phases (Fig. 1). Capybara 1 was the sole animal that was re-infected three times after primary infection (four infection challenges). Capybaras 4 and 5 had one additional infection (two infection challenges). Unexpectedly, capybaras 2 and 3 died during primary infection, impeding further exposure.

**Figure 2:**
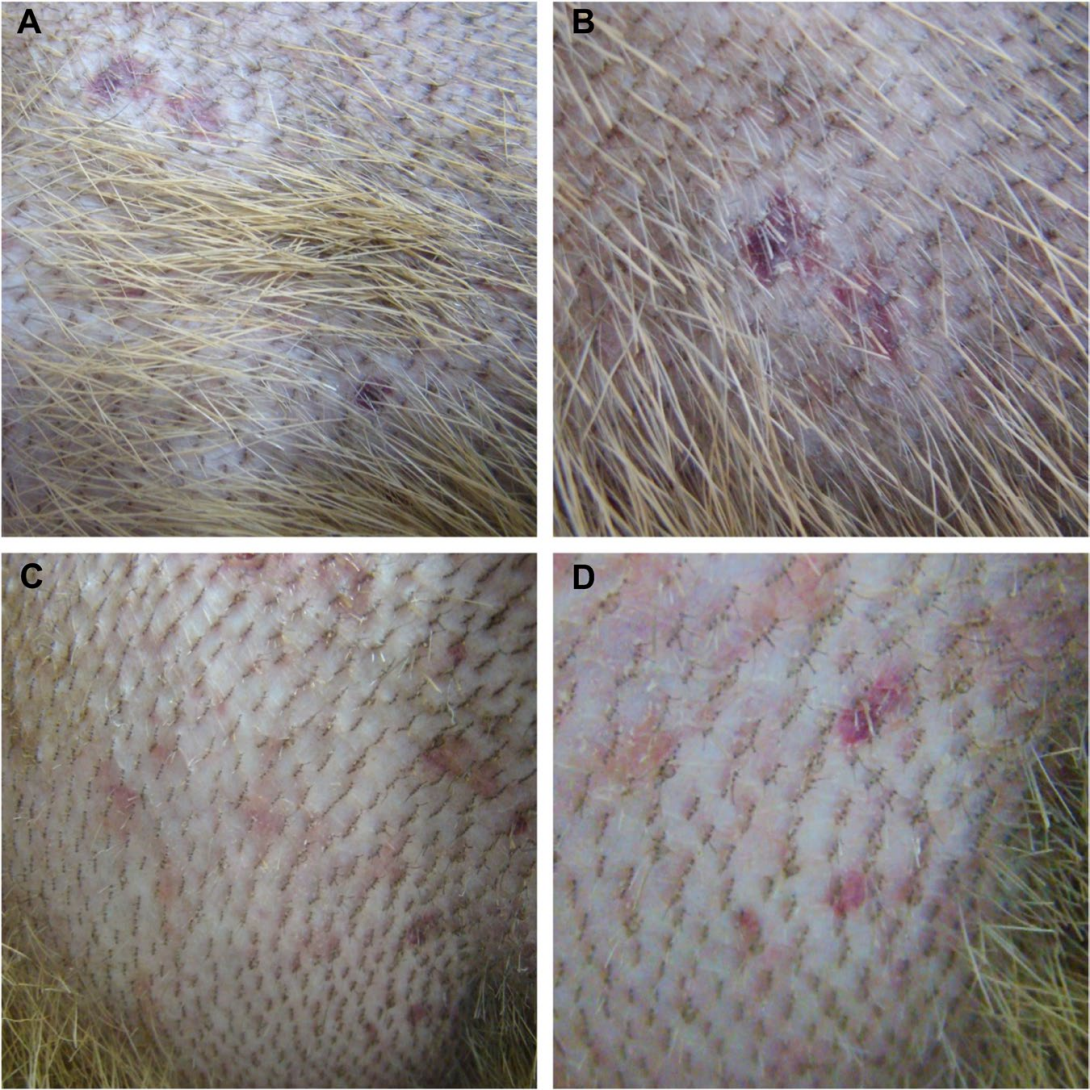
Skin of capybaras no. 2 and 3 during primary infection (phase II) with *Rickettsia rickettsii* (strain Itu) via tick exposure. (**A**,**B**) Purplish macules in capybara no. 2 (18 DPI). (**C**,**D**) Abdominal rash in capybara no. 3 (10 DPI). This figure has been published within the Doctoral Thesis of the first author (A. Ramírez-Hernández), which is available at the University of São Paulo’s digital library of Theses and Dissertations: https://teses.usp.br/teses/disponiveis/10/10134/tde-09092019-112817/en.php.

As presented in Table 1, during primary infection, capybaras 2, 3, 4 and 5 manifested rectal temperatures higher than 38.5 °C with onset on 8 or 9 DPI. Outstandingly, rectal temperature reached highest values of 39.7 and 39.8 °C in capybaras 2 and 3, respectively, which died during the bacteremic period (see below). Also, in both individuals, rectal temperatures around 32 °C (hypothermia) were recorded few hours prior to death. Capybaras 2 to 5 presented common clinical signs during primary infection, such as lack of appetite, general and hindlimb weakness (Supplementary Video), and nasal mucous discharge. Moreover, in capybaras 2 and 3, a more severe clinical course was evident with signs such as inappetence, diarrhea, dark urine, prostration, coma, seizures and death (at DPI 18 and 16, respectively). Both individuals also presented skin manifestations such as abdominal and thoracic rash and focal purplish macules with onset at 8 DPI (Table 1 and Fig. 2A–D). The first clinical signs appeared in capybaras no. 2, 3, 5 at DPI 8 and in capybara no. 4 at DPI 9 (mean incubation period: 8.3 days). In contrast, clinical alterations, including rectal temperature >38.5 °C, were absent in capybara 1 during primary infection and further challenges (phases I to IV) and in capybaras 4 and 5 during the subsequent exposure (phase IV). Also, no clinical abnormality was observed in capybara 3 when it was the uninfected control during phase I (Table 1).

**Table 1:** Clinical monitoring of five capybaras (*Hydrochoerus hydrochaeris*) during one to four exposures (experimental phases I to IV) to *Rickettsia rickettsii* strain Itu via infestations with *R. rickettsii-*infected *Amblyomma sculptum* ticks.

Capybara number	Experimental phases^#^							
	I		II		III		IV	
	Rectal Temperature*	Clinical Signs^╪^	Rectal Temperature*	Clinical Signs^╪^	Rectal Temperature*	Clinical Signs^╪^	Rectal Temperature*	Clinical Signs^╪^
1	36.7 (0.59) [35.4–37.9]	None	36.2 (0.84) [34.9–37.7]	None	36.1 (1.2) [33.4–38.0]	None	35.8 (1.05) [34.0–38.0]	None
2			36.8 (1.76) [32.0–39.7]	Abdominal and thoracic rash (8/12); fever (11/13); lack of appetite (12/14); nasal mucous discharge (13/+); hindlimb weakness (14/+); inappetence (14/+); purplish macules (16/+); prostration (18/+); coma (18/+); rigid limbs (18/+); seizures (18/+); death (18)				
3	37.1 (0.55) [35.7–38.2]	None	37.1 (1.87) [32.5–39.8]	Abdominal and thoracic rash (8/12); fever (9/13); lack of appetite (10/14); nasal mucous discharge (11/+); inappetence (14/+); hindlimb weakness (14/+); reluctance to move (15/+); diarrhea (15/+); dark urine (15/+); death (16)				
4					36.8 (0.84) [35.4–38.7]	Lack of appetite (11/15); hindlimb weakness (11/16); nasal mucous discharge (12/15); fever (13/13); general weakness (14/16)	35.8 (1.01) [34.0–37.5]	None
5					37.2 (1.13) [34.6–39.2]	Fever (8/11); mucous feces (11/16); lack of appetite (11/14)	36.3 (0.98) [33.9–38.0]	None

^#^Phase I, primary infection of capybara no. 1, and retaining capybara no. 3 as noninfected control; phase II, second infection of capybara no. 1 and primary infection of capybaras no. 2 and 3; phase III, third infection of capybara no. 1 and primary infection of capybaras no. 4 and 5; phase IV, fourth infection of capybara no. 1 and second infection of capybaras no. 4 and 5.

*Values in °C, shown as: mean (standard deviation) [range].

^╪^Clinical signs (days post infestation with infected ticks: onset/end of clinical sign).

^+^Death.

All rabbits, infested with the same batch of infected ticks used for infection challenges of capybaras, presented fever. Most of them manifested lack of appetite, auricular, preputial and scrotal vascular abnormalities (i.e. swelling, erythema or necrosis). Rabbits from phases I and II were more severely affected, manifested hypothermia and died during the acute phase of the infection; their spleen was shown to contain rickettsial DNA by real-time PCR. Rabbits from phases III and IV seroconverted to *R. rickettsii* with endpoint titres of 32,768 at 21 days after infestation.

**Figure 3:**
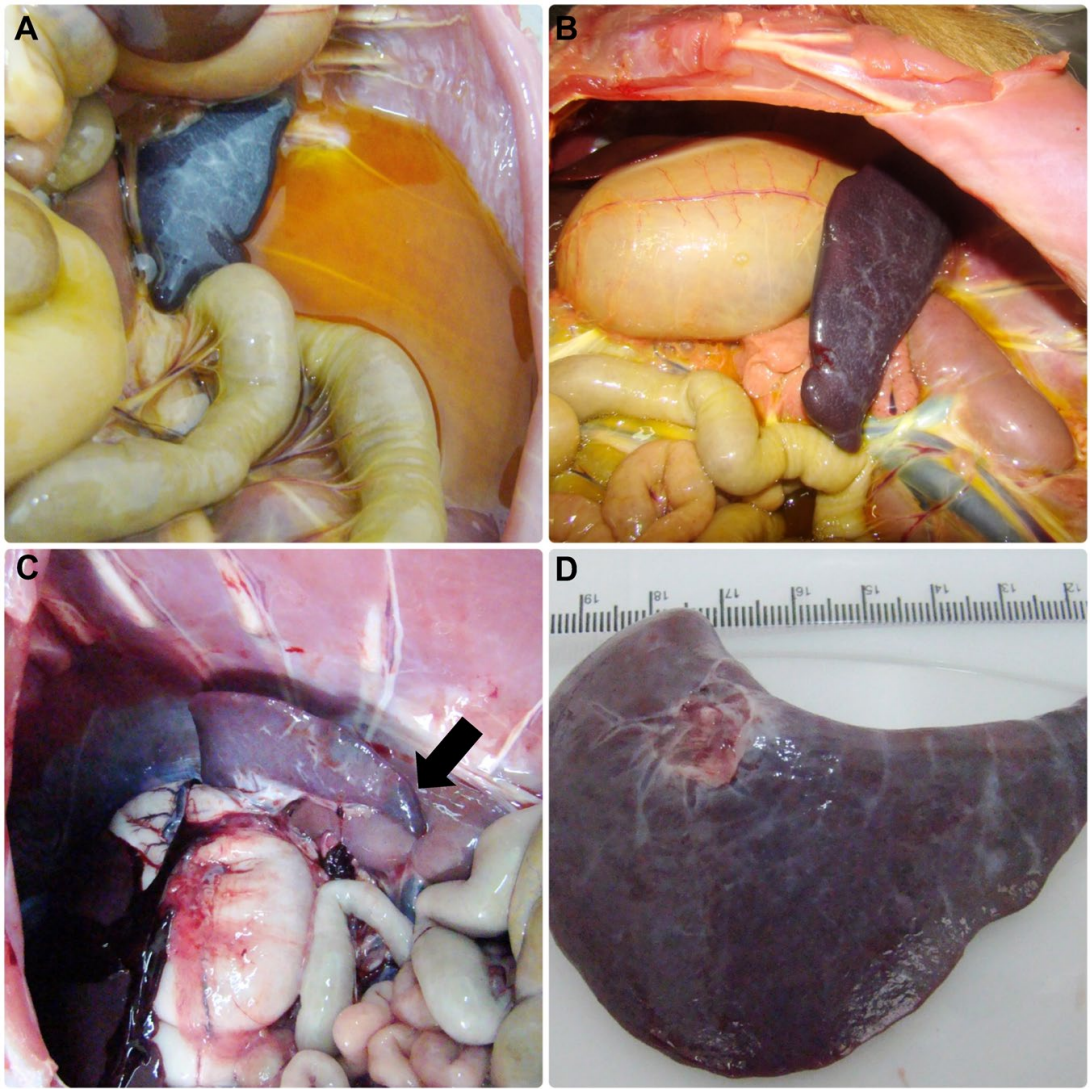
Abdominal cavity and spleen of capybaras no. 2 and 3 after primary infection (phase II) with *Rickettsia rickettsii* (strain Itu) via tick exposure. (**A**) Abdominal cavity of capybara no. 3 with evidence of ascites, jaundice and spleen enlargement (16 DPI) (**B**) Spleen enlargement in capybara no. 3 (16 DPI). (**C**) Spleen enlargement with apical hemorrhage (arrow) in capybara no. 2 (18 DPI) (**D**) Enlarged spleen from capybara no. 2 (18 DPI). This figure has been published within the Doctoral Thesis of the first author (A. Ramírez-Hernández), which is available at the University of São Paulo’s digital library of Theses and Dissertations: https://teses.usp.br/teses/disponiveis/10/10134/tde-09092019-112817/en.php.

**Figure 4:**
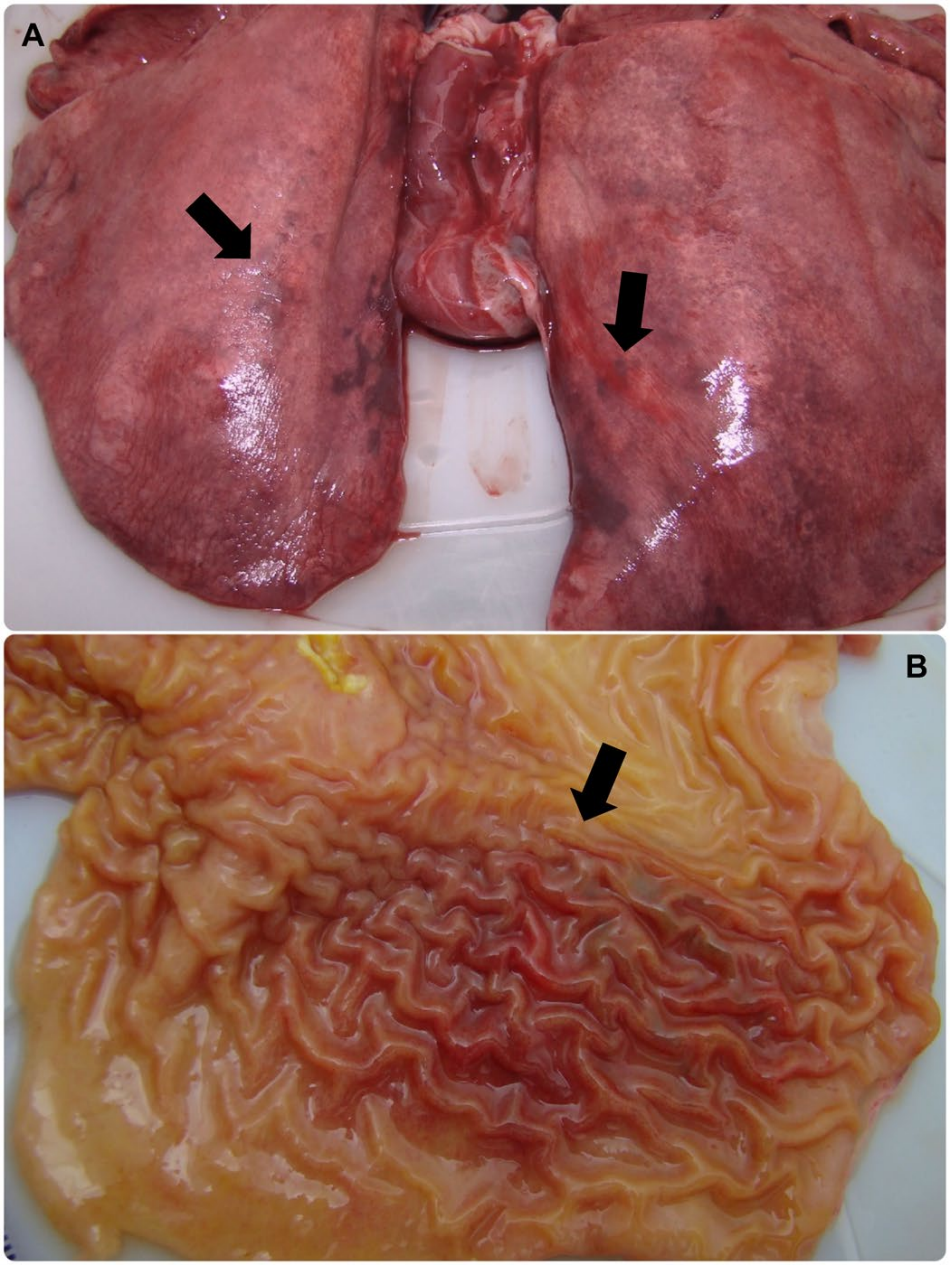
Lung and stomach of capybara no. 2 after infection (phase II) with *Rickettsia rickettsii* (strain Itu) via tick exposure. (**A**) Lung of capybara no. 2 with evidence of bilateral disseminated vascular injuries (18 DPI). (**B**) Stomach of capybara no. 2 with an extended area of hemorrhage in the mucosa (18 DPI). This figure has been published within the Doctoral Thesis of the first author (A. Ramírez-Hernández), which is available at the University of São Paulo’s digital library of Theses and Dissertations: https://teses.usp.br/teses/disponiveis/10/10134/tde-09092019-112817/en.php.

**Figure 5:**
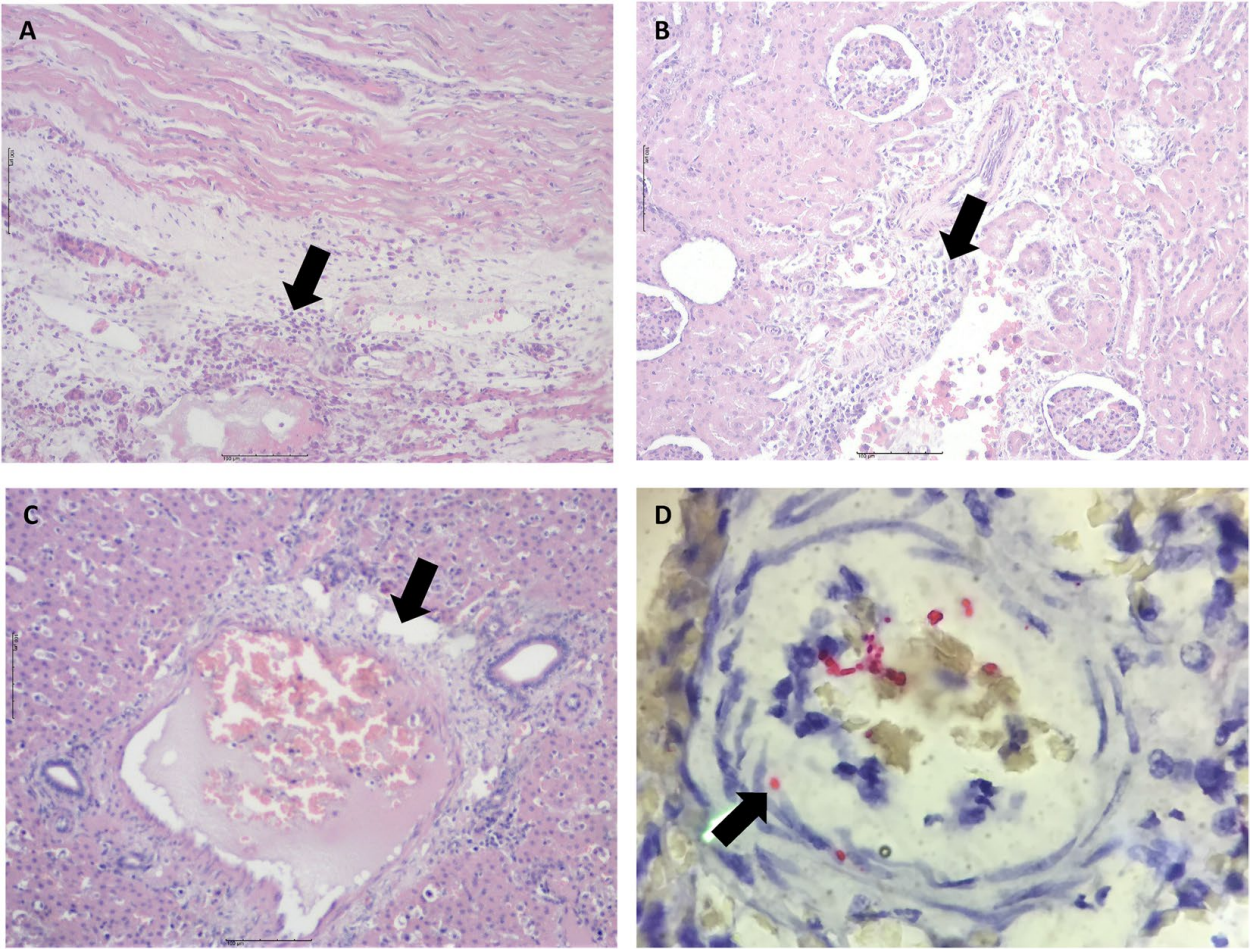
Histopathological and immunohistochemical evaluation in an experimental infection of Capybaras (*Hydrochoerus hydrochaeris*) with *Rickettsia rickettsii*. (**A**) Inflammation and vasculitis in heart (arrow) (hematoxylin and eosin staining, objective 4x). (**B)** Inflammatory cell infiltrate in kidney (arrow) (hematoxylin and eosin staining objective 4x). (**C)** Liver with vasculitis and microthrombi (arrow) (hematoxylin and eosin staining objective 10x). (**D**) Brain; immunostaining of *Rickettsia rickettsii* in vessels (red bacili) (arrow), immunoalkaline phosphatase staining, naphthol fast red substrate with hematoxylin counterstain (objective 100x).

Capybaras 2 and 3, which died during phase II, were submitted to necropsy. On gross examination, both animals exhibited disseminated lesions suggestive of vascular alterations (hyperemia and hemorrhage) in various organs such as spleen, stomach, small and large intestines, liver, lung, kidneys and adrenal glands (Figs. 3A,B, and 4). Spleen enlargement was a prominent feature in both capybaras (Fig. 3A–D). Ascites and jaundice were observed in capybara 3 (Fig. 3A). Moreover, in both capybaras, histopathology revealed a diffuse and predominant lymphohistiocytic vasculitis as well as perivascular edema in multiple tissues, including brain, spleen, gut, kidneys, heart and liver (Fig. 5A–C). Fibrin microthrombi were seen in spleen, liver and heart (Fig. 5C). Examined organs showed marked vascular congestion (spleen, liver, brain, lungs); multifocal necrosis was observed in the spleen, liver, kidneys and heart. Mixed inflammatory cellular infiltrate was observed in kidneys, heart, lungs and liver and severe emphysema in lungs. Immunohistochemical testing for rickettsiae revealed occasional staining of coccobacilli-like structures in blood vessels (Fig. 5D) from heart, spleen and brain.

### Guinea pig inoculation

Anticoagulated whole blood collected every two days from each capybara during infection challenges were inoculated into two guinea pigs simultaneously. During primary infection (first challenge) of capybaras 1 to 5, fever was detected in guinea pigs that were inoculated with blood collected from capybaras at 6 to 16 DPI, being the earliest (6 DPI) in individuals inoculated with blood from capybaras 2 and 3. The proportion of febrile guinea pigs ranged from 9.4 to 61.1% with the highest values (45.0 and 61.1%) in groups inoculated with blood from capybaras 2 and 3. None of the guinea pigs showed fever when inoculated with blood from capybaras 1, 4 and 5 during the subsequent challenges (Table 2).

**Table 2:** Clinical and serological results of guinea pigs (*Cavia porcellus*) inoculated with blood from capybaras (*Hydrochoerus hydrochaeris*) that were submitted to one to four exposures (experimental phases I to IV) to *Rickettsia rickettsii* strain Itu via infestations with *R. rickettsii-*infected *Amblyomma sculptum* ticks.

Capybara Number	Phase^#^	Guinea pig data								
		Fever*		Auricular vascular signs**	Genitalvascular signs**	Death‡		Seroconversion to *R. rickettsii*^¥^		
		No.	DPI^†^	No.	No.	No.	DPI^†^	No.	DPI^†^	Endpoint titre
1	I	3/32 (9.4)	8, 10	1/32 (3.1)	3/32 (9.4)	2/32 (6.3)	10	3/32 (9.4)	8, 12, 14	8,192
1	II	0/32 (0)		0/32 (0)	0/32 (0)	0/32 (0)		0/32 (0)		
2	II	9/20 (45.0)	6–16	10/20 (50.0)	7/20 (35.0)	4/20 (20.0)	6, 10, 16	9/20 (45.0)	6–18	262,144
3	II	11/18 (61.1)	6–16	11/18 (61.1)	9/18 (50.0)	5/18 (27.8)	6, 8, 14, 16	9/18 (50.0)	6, 10–16	131,072
1	III	0/32 (0)		0/32 (0)	0/32 (0)	0/32 (0)		0/32 (0)		
4	III	4/32 (12.5)	8–12	3/32 (9.4)	2/32 (6.3)	1/32 (3.1)	8	4/32 (12.5)	10–14	32,768
5	III	6/32 (18.8)	8–16	6/32 (18.8)	5/32 (15.6)	3/32 (9.4)	8, 14, 16	7/32 (21.9)	8–20	65,536
1	IV	0/32 (0)		0/32 (0)	0/32 (0)	0/32 (0)		0/32 (0)		
4	IV	0/32 (0)		0/32 (0)	0/32 (0)	0/32 (0)		0/32 (0)		
5	IV	0/32 (0)		0/32 (0)	0/32 (0)	0/32 (0)		0/32 (0)		

^#^Phase I, primary infection of capybara no. 1; phase II, second infection of capybara no. 1 and primary infection of capybaras no. 2 and 3; phase III, third infection of capybara no. 1 and primary infection of capybaras no. 4 and 5; phase IV, fourth infection of capybara no. 1 and second infection of capybaras no. 4 and 5.

*At least two consecutive days with rectal temperature ≥40 °C; No. refers to Number of febrile guinea pigs / total Number inoculated guinea pigs (% febrile guinea pigs).

**Vascular abnormalities like edema, erythema and/or necrosis; No. refers to Number of affected guinea pigs / total Number inoculated guinea pigs (% affected guinea pigs).

^†^DPI: days post infestation of capybara with *R. rickettsii-*infected ticks, when capybara blood was obtained and inoculated into guinea pigs.

^‡^Rickettsial DNA was amplified in spleen from all dead animals; No. refers to Number of guinea pigs that died/ total Number inoculated guinea pigs (lethality rate).

^¥^Twenty-one days after inoculation with capybara blood, guinea pigs were tested by immunofluorescence assay for seroconversion to *R. rickettsii* antigens; No. refers to Number of guinea pigs that seroconverted/ total Number inoculated guinea pigs (% seroconversion).

Auricular and genital vascular signs like erythema, edema and necrosis were recorded in guinea pigs inoculated with blood from capybaras 1 to 5 during their primary infection. The proportion of affected animals with these conditions ranged from 3.1 to 61.1%, with the highest frequencies (≥50.0%) in individuals inoculated with blood from capybaras 2 and 3. The above mentioned vascular manifestations were absent in all animals inoculated with blood from capybaras 1, 4 and 5 during subsequent challenges (Table 2).

Guinea pig death was recorded in animals inoculated with blood from capybaras 1 to 5 on 6 to 20 DPI of their primary infection. The frequency of dead animals ranged from 3.1 to 27.8%, with the highest values in groups inoculated with blood from capybaras 2 and 3. In addition, *Rickettsia* DNA was amplified from spleen collected in all dead guinea pigs. No deaths were recorded in animals inoculated with blood from capybaras 1, 4 and 5 when they were subsequently infected (Table 2).

Anti-*R. rickettsii* IgG antibodies (≥1:64 dilution) were detected by IFA in guinea pigs inoculated during primary infection of capybaras 1 to 5. The frequency of seropositive animals ranged from 9.4 to 50.0%, with highest values in animals inoculated with blood from capybaras 2 and 3. Besides, guinea pigs inoculated with 6–18 DPI-capybara blood seroconverted with endpoints titres from 8,192 to 262,144, with higher titres in animals inoculated with blood from capybaras 2 and 3 and the lowest titres in animals inoculated with blood from capybara 4. Furthermore, none of the guinea pigs inoculated with blood from capybaras 1, 4 and 5 during their subsequent challenges presented antibodies to *R. rickettsii* before and 21 days after inoculation (Table 2).

### Hematology

**Figure 6:**
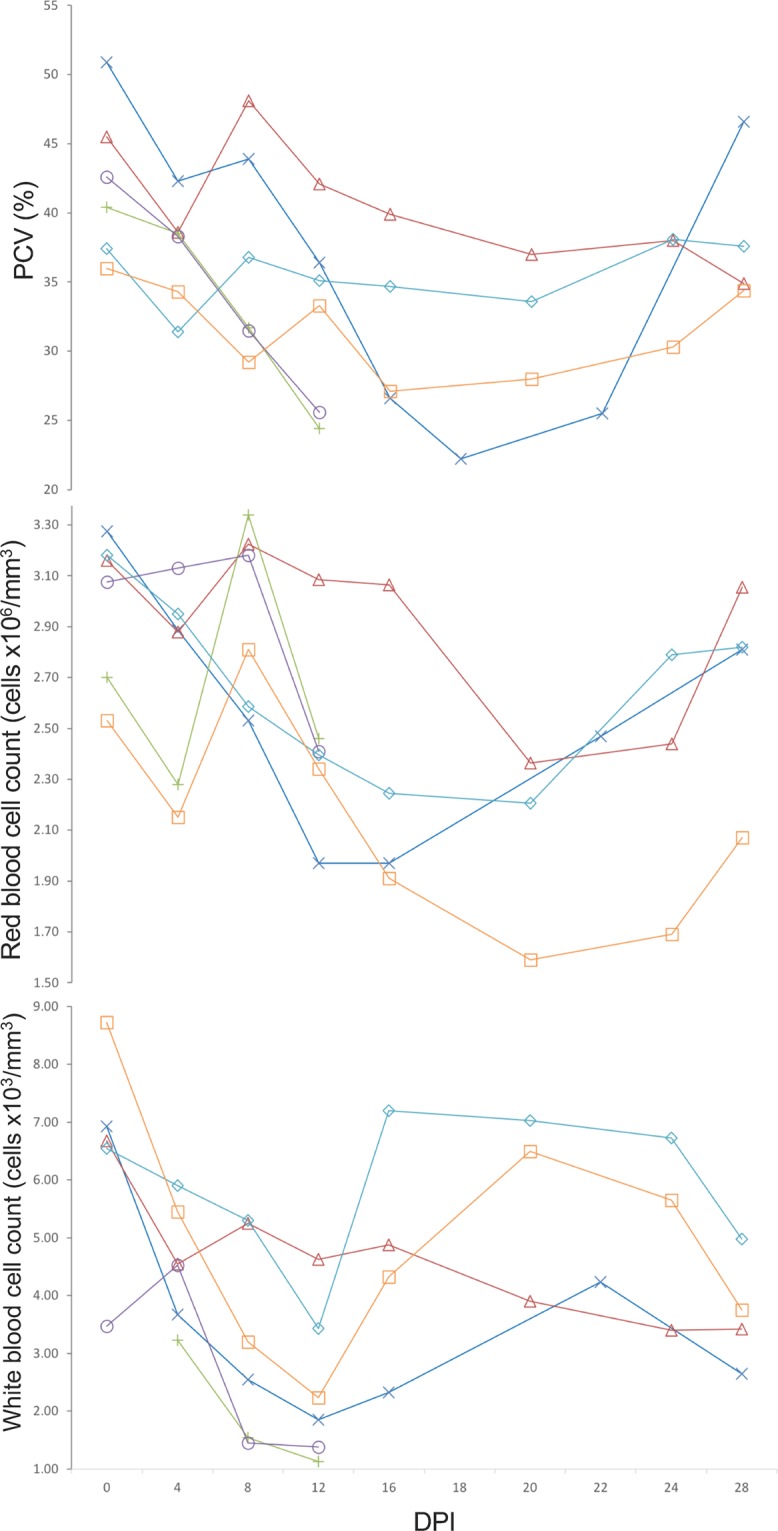
*Rickettsia rickettsii* antibody titres (IFA) after multiple infections with *R. rickettsii* (strain ITU) via tick exposures, in capybaras no. 1, 4 and 5. Dashed arrows indicate 2^nd^, 3^rd^ and 4^th^ infection of capybara no. 1 at 120, 248 and 475 days post primary infection (DPI), respectively. Straight arrow indicates 2^nd^ infection of capybaras no. 4 and 5, 227 DPI. This figure has been published within the Doctoral Thesis of the first author (A. Ramírez-Hernández), which is available at the University of São Paulo’s digital library of Theses and Dissertations: https://teses.usp.br/teses/disponiveis/10/10134/tde-09092019-112817/en.php.

Results were compared with those from capybara 3 during phase I (infested with non-infected *A*. *sculptum* ticks) and reference values (Table 3, Fig. 6). In general, mean PCV values during primary infection of all infected capybaras were lower than reference. Besides, minimum PCV values of 22.2, 24.4, 25.6, 31.4 and 27.1% in capybaras 1 to 5, respectively, were registered between 12 and 22 DPI (Fig. 6). Conversely, mean values were higher during subsequent challenges in capybaras 1, 4 and 5 (except for second infection of capybara 1) and minimum values were within the reference range (Table 3).

**Table 3:** Hematological variables evaluated in five capybaras (*Hydrochoerus hydrochaeris*) one to four exposures (experimental phases I to IV) to *Rickettsia rickettsii* strain Itu via infestations with *R. rickettsii-*infected *Amblyomma sculptum* ticks. Values presented as: mean (standard deviation) [range].

Capybara Number	Packed cell volume (%)				Red blood cell count (x10^6^ cells/mm^3^)				White blood cell count (x10^3^ cells/mm^3^)			
	Phases^#^				Phases^#^				Phases^#^			
	I	II	III	IV	I	II	III	IV	I	II	III	IV
1	36.8 (10.83) [22.2–50.9]	35.9 (2.15) [33.3–39.2]	38.7 (2.23) [35.0–41.4]	40.4 (1.49) [38.4–42.4]	2.56 (0.48) [1.97–3.28]	2.75 (0.31) [2.07–3.13]	2.66 (0.49) [1.80–3.36]	2.75 (0.18) [2.49–2.94]	3.46 (1.73) [1.85–6.93]	4.84 (0.75) [3.70–5.75]	4.72 (0.95) [3.28–6.38]	4.95 (0.64) [4.03–5.95]
2	-	33.8 (7.27) [24.4–40.4]				2.70 (0.46) [2.28–3.34]			—	1.97 (1.11) [1.13–3.23]		
3	40.5 (4.46) [34.9–48.1]	34.5 (7.49) [25.6–42.6]			2.91 (0.33) [2.37–3.23]	2.95 (0.36) [2.41–3.18]			4.59 (1.08) [3.40–6.68]	2.71 (1.56) [1.38–4.53]		
4			35.6 (2.32) [31.4–38.1]	40.6 (1.02) [39.2–41.6]	—	—	2.65 (0.35) [2.21–3.18]	3.05 (0.17) [2.78–3.32]			5.89 (1.28) [3.43–7.20]	7.62 (1.78) [5.48–10.55]
5			31.6 (3.34) [27.1–36.0]	40.8 (1.4) [38.3–42.4]	—	—	2.14 (0.42) [1.59–2.81]	3.05 (0.18) [2.81–3.29]			4.98 (2.06) [2.23–8.73]	4.94 (0.85) [3.80–6.28]
Reference values*	46.6–51.4^37^; 43.3–52.9^38^; 38.4–42.4^19^				3.44–3.98^37^; 2.82–3.44^38^; 4.3–4.7^19^				3.96–6.44^37^; 7.44–15.82^38^; 3.3–7.3^19^			

^#^Phase I, primary infection of capybara no. 1, and retaining capybara no. 3 as noninfected control; phase II, second infection of capybara no. 1 and primary infection of capybaras no. 2 and 3; phase III, third infection of capybara no. 1 and primary infection of capybaras no. 4 and 5; phase IV, fourth infection of capybara no. 1 and second infection of capybaras no. 4 and 5.

*Hematological reference values for capybaras, according to Madella *et al*.^19^, Arouca *et al*.^37^, and Van de Heijden *et al*.^38^.

Mean RBC counts in capybaras were lower than reference during primary infection of all animals, except for no. 3 (Table 3). The lowest values were registered during 12–28 DPI in all individuals including capybara 3 (Fig. 6). During subsequent challenges, mean counts of capybaras 4 and 5 were within reference values, except for capybara 1 in which values were slightly lower, in spite of being higher than the records of primary infection (Table 3).

Mean WBC counts during primary infection was lower than reference values in capybaras 2 and 3. Besides, comparing mean counts during primary infection and subsequent challenges in capybaras 1 and 4, lower values were evident during the first infection (Table 3). The minimum values were registered during primary infection of capybaras 2 and 3 (phase II), capybara 1 (phase I) and capybara 5 (phase III), with 1.13, 1.38, 1.85 and 2.23 × 10^3^ cells/mm^3^, respectively, at 12 DPI (Fig. 6).

### Serology

**Figure 7:**
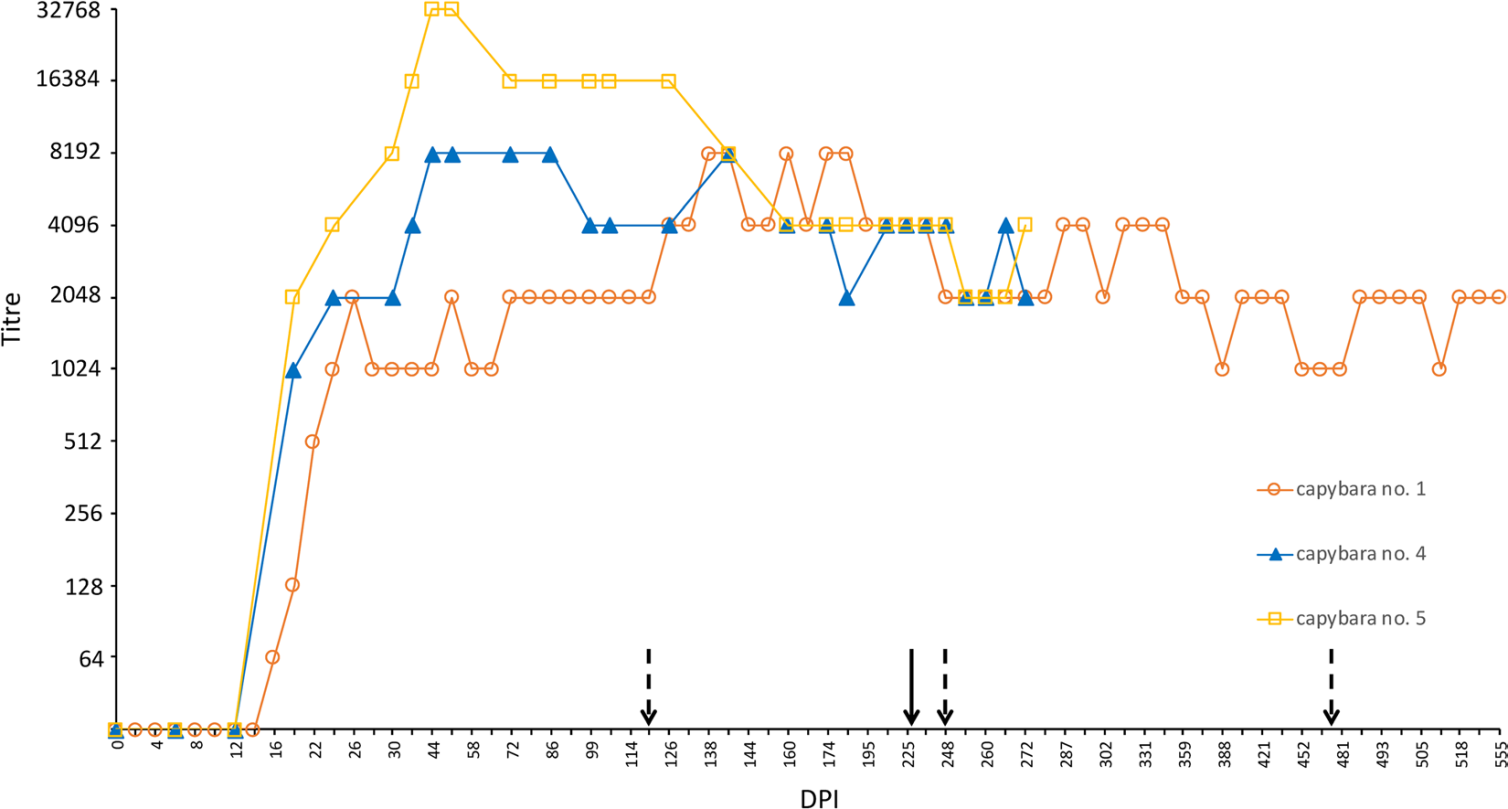
Hematological variables evaluated in capybaras (*Hydrochoerus hydrochaeris*) according to days post primary infection (DPI) with *Rickettsia rickettsii* (strain Itu) via tick exposure. Capybara numbers 1 (X), 3 (control) (△), 2 (+), 3 (○), 4 (◇) and 5 (□). This figure has been published within the Doctoral Thesis of the first author (A. Ramírez-Hernández), which is available at the University of São Paulo’s digital library of Theses and Dissertations: https://teses.usp.br/teses/disponiveis/10/10134/tde-09092019-112817/en.php.

As presented in Fig. 7, capybaras 1, 4 and 5 became first seroreactive to *R. rickettsii* at 16–18 DPI and remained seroreactive until the last day of sampling. In capybara 1, antibody titres were followed from 0 to 555 DPI. The first positive sample (≥64 titre) was registered at 16 DPI and peaked with 8,192 at 138 DPI, 18 days after the second challenge (phase II), and remained high (4,096-8,192) till 238 DPI. The lowest recorded titre was 1,024 at 388 DPI, 140 days after the third challenge. Titres at the day of the 2^nd^, 3^rd^ and 4^th^ challenges were 2,048, 2,048 and 1,024, respectively (Fig. 7). For capybara 4, the first positive sample was detected with a 1,024 titre at 18 DPI. Posteriorly, it peaked to 8,192 between 46 and 83 DPI, and thereafter, the lowest detected titre was 2,048 at 189 DPI. Before the 2^nd^ challenge (227 DPI), the titre was 4,096. For capybara 5, the first positive sample was detected at 18 DPI with a 2,048 titre, peaking to 32,768 between 46 and 54 DPI. Then, titre descended to 4,096 (159 DPI), which remained until and after the second challenge. At 251 DPI, 24 days after the second challenge, capybara 5 titre decreased to 2,048. Lastly, for capybaras 2 and 3, antibodies were detected in the serum sample before death (18 and 16 DPI, respectively) with titres of 512 and 128, correspondingly (data not graphed).

### Real-time PCR from blood and skin samples

**Figure 8:**
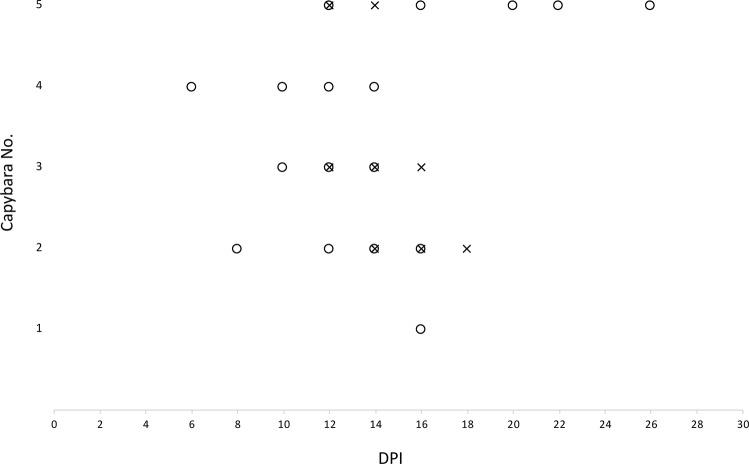
Molecular detection of *Rickettsia rickettsii* DNA in blood (X) and skin (○) samples in capybaras (*Hydrochoerus hydrochaeris*) according to days post primary infection (DPI) with *R*. *rickettsii* (strain Itu) via tick exposure.

Real-time PCR in blood samples collected during primary infection revealed *Rickettsia* DNA in samples from capybara 2 on 14, 16 and 18 DPI, capybara 3 on 12, 14 and 16 DPI, and, capybara 5 on 12 and 14 DPI. No rickettsial DNA was detected in the blood of capybaras 1 and 4 during primary infection (Fig. 8). *Rickettsia* DNA was detected in skin samples from all capybaras during primary infection, as follows: capybara no. 1 at 16 DPI; no. 2 at 8, 12, 14 and 16 DPI; no. 3 at 10, 12 and 14 DPI; no. 4 at 6, 10, 12 and 14 DPI; no. 5 at 12, 16, 20, 22 and 26 DPI (Fig. 8).

The mean number of rickettsiae per mL of blood in *R. rickettsii*-positive samples was 7.26E + 04, with the highest values recorded for capybaras no. 2 at 18 DPI (3.31E + 05) and no. 3 at 16 DPI (9.48E + 04) (Table 4). For skin samples, the mean number of rickettsia per mg of tissue was 5.24E + 02, with the highest values detected for capybaras no. 3 on 12 and 14 DPI (3.85E + 03 and 1.18E + 03, respectively) and no. 5 at 26 DPI (1.41E + 03) (Table 4).

**Table 4:** Absolute quantification of rickettsiae in *Rickettsia rickettsii*-positive samples of capybara (*Hydrochoerus hydrochaeris*) blood and skin by qPCR.

Capybara number	DPI	Number of rickettsiae per mL of blood	Number of rickettsiae per mg of skin
1	16	NA	8.84E + 01
2	8	NA	1.44E + 02
	12	NA	2.81E + 02
	14	4.12E + 04	3.04E + 02
	16	2.04E + 04	6.17E + 01
	18	3.31E + 05	NA
3	10	NA	2.17E + 02
	12	4.03E + 04	3.85E + 03
	14	2.24E + 04	1.18E + 03
	16	9.48E + 04	NA
4	6	NA	9.64E + 00
	10	NA	1.26E + 01
	12	NA	1.03E + 02
	14	NA	1.02E + 02
5	12	3.34E + 03	1.91E + 02
	14	2.69E + 04	NA
	16	NA	4.90E + 02
	20	NA	1.97E + 02
	22	NA	2.73E + 02
	26	NA	1.41E + 03

DPI: days post infestation with *R. rickettsii*-infected ticks

NA: Not analyzed because these samples did not yield rickettsial DNA in the screening qPCR analysis (see Fig. 8).

Blood samples and skin biopsies from subsequent infections were not tested because guinea pig inoculations were negative for the presence of viable rickettsia in capybara blood at all instances.

## Discussion

This study evaluated for the first time repeated experimental infections of capybaras with *R*. *rickettsii*. We tried to emulate a natural condition using ticks as the unique infection route for capybaras. Our results of guinea pig inoculations with capybara blood provided consistent evidence that capybaras developed bacteremia during the primary infection, but not during subsequent infections when they had already elicited a strong humoral response to *R. rickettsii*. In addition, rickettsial DNA was detected in the blood of three of the five capybaras (no. 2, 3 and 5) by qPCR, confirming the development of bacteremia. Only two published studies performed experimental infections of capybaras with *R. rickettsii*. The earliest work published by Travassos *et al*.^11^ performed subcutaneous inoculations of capybaras with guinea pig infected blood samples. More recently, Souza *et al*.^12^ infected capybaras by two routes, infestations with infected *A*. *sculptum* nymphs and intraperitoneal inoculation of homogenate of guinea pig infected organs. In both studies, capybaras developed bacteremia, animals were not evaluated after the primary infection, and there were no related clinical signs and fatal outcomes. In addition, the *R. rickettsii* isolates used in these previous studies were not from capybara or *A. sculptum* origin.

It is noteworthy that the *R. rickettsii* strain used in the present study (strain Itu) was previously isolated from *A. sculptum* ticks derived from a population that was sustained by capybaras under natural conditions, in Itu municipality, a BSF-endemic area of São Paulo state^16^. In addition, our tick colony of *A. sculptum* was also derived from this same BSF-endemic area. Indeed, this information supports strong field applicability of our results, especially because it was recently demonstrated that the susceptibility of *A. sculptum* to *R. rickettsii* varies among different tick populations, with a clear bias for higher susceptibility to an autochthone *R. rickettsii* strain that has already coevolved with a tick population for some time^8^.

In contrast to the present study, capybaras of the two previous studies did not present clinical alterations during the bacteremic period^11,12^. Herein we showed for the first time that capybaras manifested clinical alterations during primary infection with strain Itu of *R*. *rickettsii*. As expected, no clinical abnormality was observed in the control animal (capybara 3) infested with non-infected ticks during phase I of the study. While there are no reference values for normal rectal temperature of capybaras in the literature, in the present study we observed that rectal temperature of capybara 3 during infestation with noninfected ticks never exceeded 38.2 °C. In addition, when capybaras 1, 4 and 5 were already immune (seropositive to *R. rickettsii*) and did not develop bacteremia during subsequent challenges, their rectal temperature did not exceed 38.0 °C (Table 1). Hence, for convenience, we adopted in the present study that rectal temperatures >38.5 °C was considered as fever.

A febrile condition was manifested by capybaras 2, 3, 4 and 5 during primary infection, achieving registers as high as 39.8 °C, which differ from previous studies in which normothermic conditions were registered^11,12^. Furthermore, clinical features evidenced by febrile animals including weakness, inappetence, nasal discharge, diarrhea, nervous disorders and even death (no. 2 and 3), diverge from earlier experimental observations with absence of illness^11,12^. One possible explanation for these differences could be the *R. rickettsii* strain, which were not derived from *A. sculptum* in the two previous studies.

Regarding the afebrile condition of capybara 1, coincident with the shortest bacteremic period when compared with capybaras 2 to 5, we infer a dose-dependent condition for these differences. Before infestations, random samples of unfed adult ticks from the same batch of the ticks used for primary infection of capybaras were tested by PCR (data not shown). *Rickettsia rickettsii*-infection rates were ≈30% for ticks used in phase I (capybara 1), and 50–60% in ticks used during phases II and III (capybaras 2–5). These differences, in conjunction with the lower number of ticks used in capybara 1 (half of the ticks used in the remaining capybaras) might have contributed to the milder infection course of capybara 1. Higher infectious dose rates in capybaras 2 to 5 could be associated not only with severity of the fever and clinical course, but also with higher proportions of blood-inoculated guinea pigs presenting fever, vascular injuries, serological responses and even death. Moreover, *Rickettsia* DNA was detected in blood of only capybaras 2, 3 and 5.

Differences in infectivity, severity of clinical course, and mortality have been associated with infectious dose of *R. rickettsii* in dogs experimentally inoculated with incremental bacterial doses^23^. Furthermore, Piranda *et al*.^24^ observed differences in the severity of illness, onset of fever and bacteremia between dogs inoculated intraperitoneally and those exposed to *R*. *rickettsii* via tick bite, being more severe in the latter. Piranda *et al*.^24^ proposed that tick-exposed dogs may have received lower infectious doses over several days during adult engorgement in contrast to one intraperitoneal dose. Thus, in the present study, capybaras infested with tick batches with higher infection rates might have received higher bacterial doses during a prolonged period of tick feeding.

The clinical signs manifested by capybaras 2 to 5 during primary infection are comparable with the clinical profile of *R*. *rickettsii* infection in susceptible animal hosts (i.e. guinea pigs, rabbits, dogs and humans). As an example, fever, anorexia, lethargy, weakness, skin rash, diarrhea, prostration, seizures and death have been related in experimental and natural infections in dogs^23–26^, and are similar to those related in human cases of BSF^27,28^. Correspondingly, gross pathological lesions related to multisystemic vascular disorders observed during necropsy of capybaras 2 and 3 concur with common findings in susceptible hosts^26,29–31^. These findings were corroborated by histopathology, which revealed a multisystemic vascular injury with increased vascular permeability, inflammation, disseminated intravascular coagulation and an ultimate circulatory collapse. These observations are typical of severe acute rickettsial infection as already described for other host species including humans^26,31^ and are underscored by location of *Rickettsia* in blood vessels of capybaras by immunohistochemistry.

We relied on guinea pig inoculation with capybara blood to determine the bacteremic period in each of the infected capybaras. The mean bacteremic period was 9.2 days (range: 6–12 days). This result is similar to those reported by Souza *et al*.^12^, when the group of capybaras infected through tick infestation presented a continuous bacteremic period from 6 to 15–18 DPI (total period of 9 to 12 days). Also, they are comparable with earlier observations made by Travassos *et. al*.^11^, with bacteremia lasting from 5 to 11 DPI (6 days) in capybaras exposed by intraperitoneal inoculation. In addition, similar extents (8–12 days) were registered in diverse studies with other rodent species in the United States^9,32^. In contrast, this bacteremic period is shorter than the 26 days reported for the opossum *Didelphis aurita* in Brazil^10^, or the 4 week-period reported for *Didelphis virginiana* in the United States^33^.

In addition to referenced bacteremic periods, it is noticeable that the proportion of guinea pigs with fever, vascular clinical signs and/or seroconversion to *R. rickettsii* was higher for those animals that were inoculated with blood from capybaras 2 and 3, than in those guinea pigs inoculated with blood from capybaras 4 and 5, and even more if compared with capybara 1. Besides, highest endpoint titres (>131,072) were detected in guinea pigs inoculated with blood from capybaras 2 and 3 (Table 2). Hence, we can correlate these findings with described clinical pictures and mortality and associate them with higher bacterial loads, as proposed before.

Detection of *Rickettsia* DNA in blood was accomplished in capybaras 2, 3 and 5 between 12 and 18 DPI. This detection period coincided with the deterioration of clinical condition in these animals. Indeed, the highest loads of *Rickettsia* per mL of blood were recorded at 18 DPI for capybara no. 2 and 16 DPI for capybara no. 3, exactly when those animals died. Besides, the DNA detection time points are within the range of bacteremic period, established by guinea pig inoculation for each capybara. In the study of Souza *et al*.^12^, a single sample from one capybara yielded *Rickettsia* DNA by real-time PCR at 12 DPI. As stated before, we can infer that differences between both studies could be related to the *R. rickettsii* strain and/or the bacterial infectious doses. Regardless, it must be emphasized that techniques employing the direct detection of rickettsial DNA in bloodstream are not sensitive enough to determine bacteremic periods, especially in less severe infection courses.

*Rickettsia* DNA amplification was achieved in skin samples from all capybaras during primary infection (6–26 DPI), which is similar with those results registered by Levin *et al*.^34^ in skin biopsies from *R*. *rickettsii*-infected guinea pigs (3–22 DPI). It is remarkable that in some capybaras (no. 1 and 4), rickettsial DNA was detected in skin samples but not in blood samples. In other capybaras (no. 2, 3 and 5) skin samples yielded rickettsial DNA earlier (no. 2 and 3) or later (no. 5) than blood specimens. Similarly, Levin *et al*.^34^ detected *Rickettsia* DNA in a higher proportion of ear-skin than in blood samples from *R*. *rickettsii*-infected guinea pigs and registered earlier or later DNA detections in skin when compared with blood specimens. Those results point out the skin as an important site for rickettsial proliferation.

During primary infection of all capybaras, reduction in PCV values was perceptible for all individuals, mainly during 12–22 DPI; also, RBC counts for all capybaras, but no. 3, were below reference values during 12–28 DPI. Souza *et al*.^12^ also registered this pattern in tick-infected capybaras with decrease of PCV, RBC and hemoglobin during 15–21 DPI. Additionally, Keenan *et al*.^23^, Piranda *et al*.^24^ and Levin *et al*.^26^ also registered this hematological variation in experimentally infected dogs during the febrile period. Likewise, WBC counts reached the lowest values in capybaras 1, 2, 3 and 5 during primary infection at 12 DPI, which correlated with the febrile (for capybaras 2, 3 and 5) and bacteremic periods (all individuals). While previous studies in capybaras did not include WBC count, these results are comparable with those described by Keenan *et al*.^23^ in experimental dogs. However, leukocytosis was a more common pattern in infected animals, explained mainly by predominant monocyte and granulocyte responses (monocytosis and granulocytosis)^26^. Lastly, similar studies must perform thrombocyte counts due to its variability in infected hosts^35^. Herein, lack of standardization in manual counts and in blood smear estimation for capybaras precluded reliable thrombocyte counts.

Quantification of anti-*R*. *rickettsii* IgG antibodies through IFA indicates a strong humoral response in all capybaras. First reactive samples were detected between 16 and 18 DPI and then peaked heterogeneously (26–46 DPI) among convalescent individuals (capybaras 1, 4 and 5) and presented a variable dynamic until the end of the study (307–555 days). Maximum titres were 8,192 (animals 1 and 4) and 32,768 (no. 5). It is noteworthy that capybara 1-highest titre (8,192) was recorded after the 2^nd^ challenge (phase II), when death of capybaras 2 and 3 was noted. Capybara 1’s previous highest endpoint (2,048) was observed at 26 DPI (phase I). A probable strong antigenic stimulus could explain titre raise in capybara 1 after the second infection, a phenomenon not evidenced in capybaras 4 and 5. IFA results agree with those of Souza *et al*.^12^ in which capybaras infected through tick feeding showed the first detectable humoral response at 12 DPI and then peaked from 21 to 30 DPI, with mean endpoint titres varying from 8,192 to 32,768, which remained till the end of the following period (146 DPI).

One main objective of the present study was to evaluate clinical, hematological, immunological and infectious variables in capybaras after subsequent challenges with *R*. *rickettsii* through tick feeding. Data was collected in capybara 1 during three subsequent challenges at 120, 248 and 475 DPI. Besides, for capybaras 4 and 5, data was obtained for one subsequent challenge at 227 DPI. As stated before, none of the animals presented fever, clinical signs or hematological abnormalities during subsequent infections. Also, guinea pigs inoculated with blood during these phases did not reveal fever, clinical signs or specific antibody responses. Moreover, through antibody follow-up period, titres ranging from 1,024 to 4,096 were noticeable, previous to each challenge, corroborating a sustained immune response in capybaras, even without recent antigenic stimuli (the maximum interval between challenges was 227 days). Thus, we can infer that primarily infected capybaras develop a durable protective immune response that counteract further *R*. *rickettsii* challenges and impeded its corporal dissemination and establishment. Likewise, Keenan, *et al*.^23^ conducted an experimental challenge of convalescent dogs with high *R*. *rickettsii* doses, six and twelve months after primary infection, and found no clinical nor hematological abnormalities.

One drawback of this study could be that the number of *R. rickettsii-*infected ticks used for primary infection of capybaras could be much higher than those to which capybaras are usually exposed under natural conditions. This assumption relies on the fact that ≤1% of the *A. sculptum* ticks is usually found infected by *R. rickettsii* under natural conditions in BSF-endemic areas^3,16,21^. However, in our study, capybaras were heathy and at good nutrition before primary infection, what could be major factors contributing to the less severe clinical outcome of capybara no. 1, which received the least number of infected ticks in this study. On the other hand, if concomitant diseases and malnutrition are factors contributing to a more severe outcome of *R. rickettsii-*primary infection of capybaras under natural conditions, it is yet to be investigated.

In conclusion, the present study confirms that capybaras are susceptible to the strain Itu of *R*. *rickettsii*, isolated from an *A*. *sculptum* population of capybara origin. In addition, this susceptibility could be dose-dependent due to evidence of fever, illness and mortality in some of the infected animals that received higher number of infected ticks. Bacteremic period, hematologic and serologic patterns are similar to those reported previously by experimental capybara infection with a *R*. *rickettsii* strain derived from *A*. *aureolatum* ticks (Taiaçu). Finally, it is confirmed, under experimental conditions, that infected capybaras develop a sustained immune response that prevents a further bacteremia. This condition may imply a high reproduction rate of capybaras in BSF-endemic areas, in order to continuously generate capybaras susceptible to bacteremia during primary infection. This statement relies on the fact that *A. sculptum* ticks are partially refractory to *R. rickettsii*, and in the absence of vertebrate amplifying hosts (e.g. capybaras in bacteremia), *R. rickettsii* would disappear from the *A. sculptum* population after a few tick generations^36^.

## Supplementary information

Supplementary video caption.

Supplementary video.

## Data Availability

The data from this study are available from the corresponding author upon reasonable request.
