# Author Correction: Clinical and serological evaluation of capybaras (*Hydrochoerus hydrochaeris*) successively exposed to an *Amblyomma sculptum*-derived strain of *Rickettsia rickettsii*

**DOI:** 10.1038/s41598-020-61342-2

**Published:** 2020-03-11

**Authors:** Alejandro Ramírez-Hernández, Francisco Uchoa, Maria Carolina de Azevedo Serpa, Lina C. Binder, Alessandra Castro Rodrigues, Matias P. J. Szabó, Andrea Fogaça, Celso Eduardo Souza, Marcelo B. Labruna

**Affiliations:** 1 Department of Preventive Veterinary Medicine and Animal Health, Faculty of Veterinary Medicine, University of São Paulo, Av. Prof. Orlando Marques de Paiva 87, São Paulo, SP 05508-270 Brazil; 2 Reference Rickettsial Diseases Laboratory, Superintendence for Control of Endemic Diseases, Mogi Guaçu, SP Brazil; 3 Ixodology Laboratory, Faculty of Veterinary Medicine, Federal University of Uberlândia, Uberlândia, MG Brazil; 4 Department of Parasitology, Institute of Biomedical Sciences, University of São Paulo, São Paulo, SP Brazil

Correction to: *Scientific Reports* 10.1038/s41598-020-57607-5, published online 22 January 2020

This Article contains an error in the Figure legends of Figure 6 and Figure 7. The legends of these Figures were inadvertently switched.

The legend of Figure 6:

“Rickettsia rickettsii antibody titres (IFA) after multiple infections with R. rickettsii (strain ITU) via tick exposures, in capybaras no. 1, 4 and 5. Dashed arrows indicate 2nd, 3rd and 4th infection of capybara no. 1 at 120, 248 and 475 days post primary infection (DPI), respectively. Straight arrow indicates 2nd infection of capybaras no. 4 and 5, 227 DPI. This figure has been published within the Doctoral Thesis of the first author (A. Ramírez-Hernández), which is available at the University of São Paulo’s digital library of Theses and Dissertations: https://teses.usp.br/teses/disponiveis/10/10134/tde-09092019-112817/en.php.”

should read:

“Hematological variables evaluated in capybaras (Hydrochoerus hydrochaeris) according to days post primary infection (DPI) with Rickettsia rickettsii (strain Itu) via tick exposure. Capybara numbers 1 (X), 3 (control) (Δ), 2 (+), 3 (◯), 4 (◇) and 5 (□). This figure has been published within the Doctoral Thesis of the first author (A. Ramírez-Hernández), which is available at the University of São Paulo’s digital library of Theses and Dissertations: https://teses.usp.br/teses/disponiveis/10/10134/tde-09092019-112817/en.php.”

The legend of Figure 7:

“Hematological variables evaluated in capybaras (Hydrochoerus hydrochaeris) according to days post primary infection (DPI) with Rickettsia rickettsii (strain Itu) via tick exposure. Capybara numbers 1 (X), 3 (control) (Δ), 2 (+), 3 (◯), 4 (◇) and 5 (□). This figure has been published within the Doctoral Thesis of the first author (A. Ramírez-Hernández), which is available at the University of São Paulo’s digital library of Theses and Dissertations: https://teses.usp.br/teses/disponiveis/10/10134/tde-09092019-112817/en.php.”

should read:

“Rickettsia rickettsii antibody titres (IFA) after multiple infections with R. rickettsii (strain ITU) via tick exposures, in capybaras no. 1, 4 and 5. Dashed arrows indicate 2nd, 3rd and 4th infection of capybara no. 1 at 120, 248 and 475 days post primary infection (DPI), respectively. Straight arrow indicates 2nd infection of capybaras no. 4 and 5, 227 DPI. This figure has been published within the Doctoral Thesis of the first author (A. Ramírez-Hernández), which is available at the University of São Paulo’s digital library of Theses and Dissertations: https://teses.usp.br/teses/disponiveis/10/10134/tde-09092019-112817/en.php.”

