# Supplementary material for: Clinical and serological evaluation of capybaras (Hydrochoerus hydrochaeris) successively exposed to an Amblyomma sculptum-derived strain of Rickettsia rickettsii
Source: Sci Rep. 2020 Jan 22;10:924. doi: 10.1038/s41598-020-57607-5 (PMC6976648; doi:10.1038/s41598-020-57607-5)
Supplement: Supplementary file 1 — Supplementary video caption. [file 41598_2020_57607_MOESM1_ESM.docx]

**MANUSCRIPT TITLE: Clinical and serological evaluation of capybaras (*Hydrochoerus hydrochaeris*) successively exposed to an *Amblyomma sculptum*-derived strain of *Rickettsia rickettsii***

Alejandro Ramírez-Hernández,^1^ Francisco Uchoa,^2^ Maria Carolina de Azevedo Serpa,^1^ Lina C. Binder,^1^ Alessandra Castro Rodrigues,^3^ Matias P. J. Szabó,^3^ Andrea Fogaça,^4^ Celso Eduardo Souza,^2^ Marcelo B. Labruna^1*^

^1^ Department of Preventive Veterinary Medicine and Animal Health, Faculty of Veterinary Medicine, University of São Paulo, Av. Prof. Orlando Marques de Paiva 87, São Paulo, SP, 05508-270, Brazil

^2^ Reference Rickettsial Diseases Laboratory, Superintendence for Control of Endemic Diseases, Mogi Guaçu, SP, Brazil

^3^ Ixodology Laboratory, Faculty of Veterinary Medicine, Federal University of Uberlândia, Uberlândia, MG, Brazil

^4^ Department of Parasitology, Institute of Biomedical Sciences, University of São Paulo, São Paulo, SP, Brazil

*Correspondence and requests for materials should be addressed to M.B.L.

(email: [labruna@usp.br](mailto:labruna@usp.br))

**Supplementary video caption:** Video showing hindlimb weakness in capybara no. 2 during primary infection with *Rickettsia rickettsii* strain Itu at 14 days post infestation with infected ticks.
